# COVID-19 vaccine uptake among physicians during the second wave of COVID-19 pandemic: Attitude, intentions, and determinants: A cross-sectional study

**DOI:** 10.3389/fpubh.2022.823217

**Published:** 2022-08-03

**Authors:** Samar A. Amer, Jaffer Shah, Eman Elsayed Abd-Ellatif, Hala A. El Maghawry

**Affiliations:** ^1^Department of Public Health and Community Medicine, Faculty of Medicine Zagazig University, Zagazig, Egypt; ^2^Member of the Royal College of General Practitioners, London, United Kingdom; ^3^Mental Health in Primary Care, Nova University, Lisbona, Portugal; ^4^New York State Department of Health, New York, NY, United States; ^5^Department of Public Health and Community Medicine, Faculty of Medicine, Mansoura University, Mansoura, Egypt; ^6^Department of Public Health and Community Medicine, Faculty of Medicine Zagazig University, Zagazig, Egypt

**Keywords:** vaccination, COVID-19, attitude - intentions, cross sectional analysis, physician, COVID-19 Vaccination

## Abstract

**Background:**

Developed within a short period of time, the COVID-19 vaccine is not yet widely accepted among the public despite its availability, including by physicians, who are considered a vulnerable group.

**Methodology:**

A descriptive cross-sectional study selected 436 governmental physicians from different specializations, representing four random administrative regions in Egypt. The data were collected through a self-administrated online questionnaire and analyzed using suitable tests.

**Results:**

Out of the studied 436 physicians, 229 (52.2%) [aged 20–30, 284 (65.1%)] were women, 270 (61.9%) were residents, 219 (50.2%) were married, 398 (91.3%) were non-smokers, and 263 (60.3%) were non-frontline caregivers. The majority of the physicians, 227 (52.1%) of them, hesitated to take the vaccine, 236 (54.1%) had not decided on the preferred type of vaccine, and 101 (23.2%) were neutrally confident in the Egyptian healthcare system; 302 (96.3%) had no history of drug or food hypersensitivity. There was no statistically significant (*p* < 0.05) relationship between the physicians' attitude toward COVID-19 vaccine uptake and the studied demographic variables. There was a statistically significant connection between all of the doctors' intentions to get the COVID-19 vaccine and all of the four attitude domains that were looked at.

**Conclusion:**

The study concluded that a low level of willingness among Egyptian physicians to take the COVID-19 vaccine is a prevalent problem. COVID-19 vaccination hesitancy and non-acceptance were linked to negative attitudes about natural immunity, mistrust of vaccine benefits, and concerns about commercial profiteering.

## Introduction

The World Health Organization (WHO) has made stringent efforts and campaigns to advise the world on how to manage and overcome the COVID-19 pandemic (1). There are no specific antiviral medications and only a few drugs have shown the potential to reduce mortality among COVID-19 patients (2). As a result, human compliance with global preventive measures, such as facemasks, social distancing, extended quarantine, and travel restrictions, has been shown to only go so far in reducing the spread of the virus. The best way to control and eventually eradicate this pandemic is to produce an effective and accessible vaccine (3).

Vaccination is one of the biggest public health successes of the 20th century, with at least seven COVID-19 vaccines having been developed as of 18 February 2021. High vaccination coverage is advised as the main public health intervention to control and flatten the epidemic “curve” of COVID-19, with the long-term goal of achieving herd immunity; however, in most cases, vaccine development can take years. As a result, despite the availability of the new COVID-19 vaccine, public acceptance remains uncertain (4). Vaccine hesitancy is one of the most difficult health challenges, so much so that the WHO considers it a significant global health threat (5).

Several factors influence the decision to accept, postpone, or refuse vaccination, including political, cultural, ecological, healthcare system, historical, and socioeconomic factors (6). According to Protection Motivation Theory, factors such as vaccination perceptions, efficacy, the severity of health threats, and a low incidence of community infections can influence a person's willingness to get vaccinated, making them important tenets of health behavior engagement. In particular, concerns about side effects or safety, as well as the social and peer factors can heavily influence a person's willingness to get vaccinated (7, 8).

Misinformation, safety or efficacy concerns, the vaccine manufacturer's country of origin, and the belief in rushed vaccine development and production are the main causes of COVID-19 vaccination hesitancy (6). It has also been observed that specific vaccine-related issues, such as new vaccine introduction, administration method, schedule, cost, reliability, source of supply, knowledge base, new recommendations for a current vaccine, and the strength of these recommendations also play a part in the public's hesitation to take the vaccine. Globally, the acceptance rate of the COVID-19 vaccine varies and has been linked to some of these factors in many studies. The Middle East, in particular, has been one of the regions with the lowest rates of vaccine acceptance (7).

Developing effective COVID-19 vaccination strategies requires a thorough study of the factors that influence vaccination decisions as they might differ significantly between people who accept and are determined to take the vaccine and those who do not (9). According to a recent global report, approximately 30% of those polled would refuse or are hesitant to take the vaccine if it becomes available (10).

The role of healthcare providers (HCPs) in the pandemic response has become increasingly important. Due to low vaccination acceptance rates among HCPs, individuals who deal with vaccine-hesitant HCPs both professionally and personally are likely to be less vaccine-compliant. This is concerning because HCPs are the most dependable social resource for promoting public immunization, as they are in the best position to comprehend and respond to the anxieties and concerns of hesitant patients, as well as to explain the benefits of vaccination, especially during subsequent waves of COVID-19 (11–13).

Only a few studies among HCPs have been conducted to address these issues. HCPs who are exposed to COVID-19 patients are at risk of contracting the virus and transferring it to others (14). Therefore, achieving high COVID-19 vaccination coverage rates for this group will be paramount, as they are considered immunization role models for the public and have substantial influence over individuals and their communities. They will also be responsible for recommending vaccinations and counseling COVID-19-positive patients (15).

Limited research has studied COVID-19 vaccine acceptance among HCPs in Egypt during the second wave of the COVID-19 pandemic. At that time, vaccine availability was restricted in Egypt, a middle-income country in northeast Africa. Vaccination campaigns had not yet been initiated, and only HCPs were eligible for vaccination. The goal of this study was to investigate physicians' attitudes and acceptance of the COVID-19 vaccine, as well as the determinants that may influence their vaccination decision-making from January to March 2021.

## Methodology

### Participants and study design

This online cross-sectional survey targeted Egyptian physicians of different specialties and was conducted from January 2021 to March 2021. The exclusion criteria were refusal to participate in the study, internet non-users, and Egyptian physicians living or working abroad during the study period.

### Sample size

The sample size was estimated using this equation: n = Z^2^ P (1 – P) / d^2^ (16).

*n* = sample size, *z* = level of confidence according to the standard normal distribution (for a level of confidence of 95%, *z* = 1.96, for a level of confidence of 99%, *z* = 2.575), *P* = estimated proportion of the population that presents the characteristic (when unknown, we use *P* = 0.5), *d* = tolerated margin of error (for example, we want to know the real proportion within 5%) (16). Due to limited data regarding the prevalence of COVID-19 anti-vaccine attitudes in Egypt, we assumed that 50% of the respondents would have anti-COVID-19 vaccine attitudes, 95% confidence level, and 80% power of the study, so the calculated sample size was 436 physicians.

### Sampling techniques

The data were anonymously collected using a multistage sampling method *via* an online self-administered questionnaire. We randomly selected four of Egypt's seven geographical regions, then randomly selected four governmental healthcare settings per region, two from urban areas and two from rural areas (17). The targeted sample from each was weighted according to proportions based on physician density per setting.

### Data collection

Google Forms was used to create, distribute, and collect the questionnaire. The data were gathered using a self-administered online English questionnaire. From January through March 2021, the URL was shared *via* the study team's network and the HCPs' professional groups, as well as the official platforms of many healthcare settings on WhatsApp, Facebook, official emails, and Facebook Messenger. Data confidentiality was guaranteed. A weekly reminder was sent to increase the response rate until the target sample was reached.

Data were collected anonymously through an online survey based on another study (18). The questionnaire was revised and then pilot tested on 15 HCPs to check acceptability, clarity, and face validity. The results of the pilot study were not used in the final analysis. Internal consistency was assessed, and Cronbach's coefficient was 0.82. The questionnaire contained required admission of sensitive information.

### Data collection tool

The questionnaire was composed of four main sections as follows:

1. Sociodemographics
2. Age, sex, residence, educational level, frontline physician status, experience in years, marital status, smoking history, and history of chronic diseases.
3. COVID-19 exposure history and health-related factors

- Previous infection with COVID-19.
- Family member infected with COVID-19.
- Perceived susceptibility to COVID-19 infection.
- General Health Perception Scale; single item to determine the current perceived state of health (poor to medium health, good health, and very good to excellent health) 0.1 (2).
- Confidence in the Egyptian government to handle the pandemic.

*3. COVID-19 vaccine uptake-related factors*

□ The preferred type of vaccine.
□ Willingness (willing, hesitated, or unwilling) to take the vaccine.
□ History of potential adverse effect or sensitivity to food or drug or medication , using a single item based on the perceived sensitivity to medication scale (19).
□ History of influenza vaccination.

### 4-vaccine attitude

The 12-item Vaccination Attitudes Examination (VAX) Scale (20) was used to assess four negative attitudes toward the COVID-19 vaccine with subscale items: worries about unforeseen side effects, natural immunity preference, mistrust of vaccine benefits, and commercial profiteering concerns.

Each item was assessed through a six-point Likert scale ranging from strongly agree = 1; agree = 2; slightly agree =3; neutral = 4; slightly disagree = 5; to strongly disagree = 6. The total scores ranged from 6 to 24; the higher the score, the more negative the attitude. Values equal to or above the mean of the total score or individual items were considered negative attitudes of the participants toward the vaccine, while those below the mean were considered positive attitudes.

### Statistical analysis

The data were analyzed using SPSS version 25 (IBM, Armonk, NY, United States). Differences were considered statistically significant at *p* < 0.05. The qualitative and discrete sociodemographic variables were presented as frequency and percentage. A chi-square test was performed to test the relationship between sociodemographic factors and COVID-19 vaccination uptake. The mean and standard deviation were used to calculate the quantitative subscales of attitudes toward vaccinations. Pearson's correlation coefficient (r) was used to test the association between age and the subscales of the VAX Scale. The predictors of COVID-19 vaccination uptake among physicians, hesitancy, and non-acceptance were identified using multinomial logistic regression analysis.

### Ethical issues

The study methodology was approved by the Ethical Committee of Scientific Research, Faculty of Medicine, Benha University, No. RC.3.1.2021. All participants provided electronic informed written consent after clarification of the goals, data confidentiality, voluntary participation, and withdrawal.

## Results

Out of the 436 physicians studied, 229 (52.2%) were between the ages of 20 and 30 years, 239 (54.8%) lived in an urban area, 284 (65.1%) were women, 270 (61.9%) were residents, 219 (50.2%) were married, 398 (91.3%) were non-smokers, 263 (60.3%) were not frontline caregivers, and 260 (59.6%) had fewer than 10 years of experience (Table 1).

**Table 1:** Association between sociodemographic and attitude toward COVID-19 vaccine uptake.

**Sociodemographic** **variables**	**Total *n* = 436** ***F* (%)**	**Attitude**		***X*^2^ test (*p*)**
		**Positive** **No=222 *F* (%)**	**Negative** **No=214 *F* (%)**	
**Age groups (Y)**				
20– <30	229(52.2)	120(54.1%)	109(50.9%)	1.25
30– <40	150(34.4)	71(31.9%)	79(36.9%)	(0.56)
40 y or more	57(13.1)	31(13.9%)	26(12.1%)	
**Residence**				
Urban	239 (54.8)	120(54.1%)	119(55.6%)	0.12
Rural	197(45.2)	102(45.9%)	95(44.4%)	(0.77)
**Sex**				
Male	152(34.9)	71(31.9%)	81 (37.9%)	1.65
Female	284(65.1)	151(68.0%)	133(62.1%)	(0.23)
**Education**				
Resident (University-educated)	270(61.9)	139(62.6%)	131(61.2%)	0.09
Post graduate	166(38.1)	83(37.3%)	83(38.8%)	(0.76)
**Marital status**				
Single	205(47.0)	109(49.1%)	96(44.9%)	2.02
Married	219(50.2)	109(49.1%)	110(51.4%)	(0.36)
Divorced –widowed	12(2.8)	4(1.8%)	8(3.7%)	
**Smoking**				
Current smoker	23(5.3)	11(4.9%)	12(5.6%)	0.124
Ex-smoker	15(3.4)	8(3.6%)	7(3.3%)	(0.96)
Non-smoker	398(91.3)	203(91.4%)	195(91.1%)	
**Frontline**				
Yes	173(39.7)	90(40.5%)	83(38.8%)	0.14
No	263(60.3)	132(59.4%)	131(61.2%)	(0.77)
**Experience duration (y)**				
≤ 10 y	260(59.6)	130(58.6%)	130(60.7%)	0.22
>10 y	176(40.4)	92(41.4%)	84(39.3%)	(0.69)

X^2^ test (chi-square test).

In terms of health-related factors, the majority of the physicians studied (336, 77.1%) had no comorbidities, 261 (59.9%) had never received a flu vaccine, 229 (52.5%) were not infected with COVID-19, 336 (77.1%) reported that they were susceptible to infection, and 192 (44.0%) had no family members infected with COVID-19 (Table 2).

**Table 2:** Association between the physicians' attitude toward COVID-19 vaccine uptake and health-related factors.

**Health-related** **variables**	**Total *n* = 436** ***F* (%)**	**Attitude**		**X^2^ test (*p*)**
		**Positive attitude** **No=222 *F* (%)**	**Negative attitude** **No=214 *F* (%)**	
**Health status**				
Poor to medium health	31(7.1)	9(4.1%)	25(11.7%)	9.36
Good health	133(30.5)	67(30.2%)	66(30.8%)	(0.009) ^*^
Very good to excellent	194(44.5)	146(65.8%)	123(57.5%)	
**Chronic disease**				
No chronic disease	336(77.1)	169(76.1%)	167(68.1%)	0.23
With comorbidities ^**^	110(22.9)	53(23.9%)	47(21.9%)	(0.65)
**Up took flu vaccine**				
Never take flu vaccine	261 (59.9)	133(59.9%)	128(59.8%)	
Took flu vaccine before^***^	157(40.1)	89(40.1%)	86(40.1%)	
**Get infected with COVID-19**				
No	95(21.8)	52(23.4%)	43(20.1%)	4.86
Yes	229(52.5)	123(55.4%)	106(49.5%)	(0.09)
Don't know	112 (25.7)	47(21.7%)	65(30.4%)	
**Perceived susceptibility**				
Yes, I'm susceptible	336(77.1)	161(72.5%)	175(81.8%)	7.27
No, I don't susceptible	23(5.2)	17(7.6%)	6(2.8%)	(0.027) ^*^
Don't know	77(17.7)	44(19.8%)	33(15.4%)	
**Family with COVID-19**				
Yes	184(42.2)	97(43.7%)	87(40.7%)	0.42
No	192(44.0)	95(54.8)	97(45.3%)	(0.82)
Don't know	60(13.8)	30(13.5%)	30(14.2%)	

* Significant at the 0.05 level (2-tailed). X^2^ test (chi-square test).

** Asthma or respiratory disease 22 (5.0), cardiac disease 4 (0.9), hypertension 11 (2.5), diabetes 11 (2.5), kidney or liver disease 2 (0.5), autoimmune disease 9 (2.1), overweight/obesity 21 (4.8).

*** Long time ago100 (22.9), last year 12 (2.8), this year 44 (10.1), and every year 19 (4.4).

**Figure 1:**
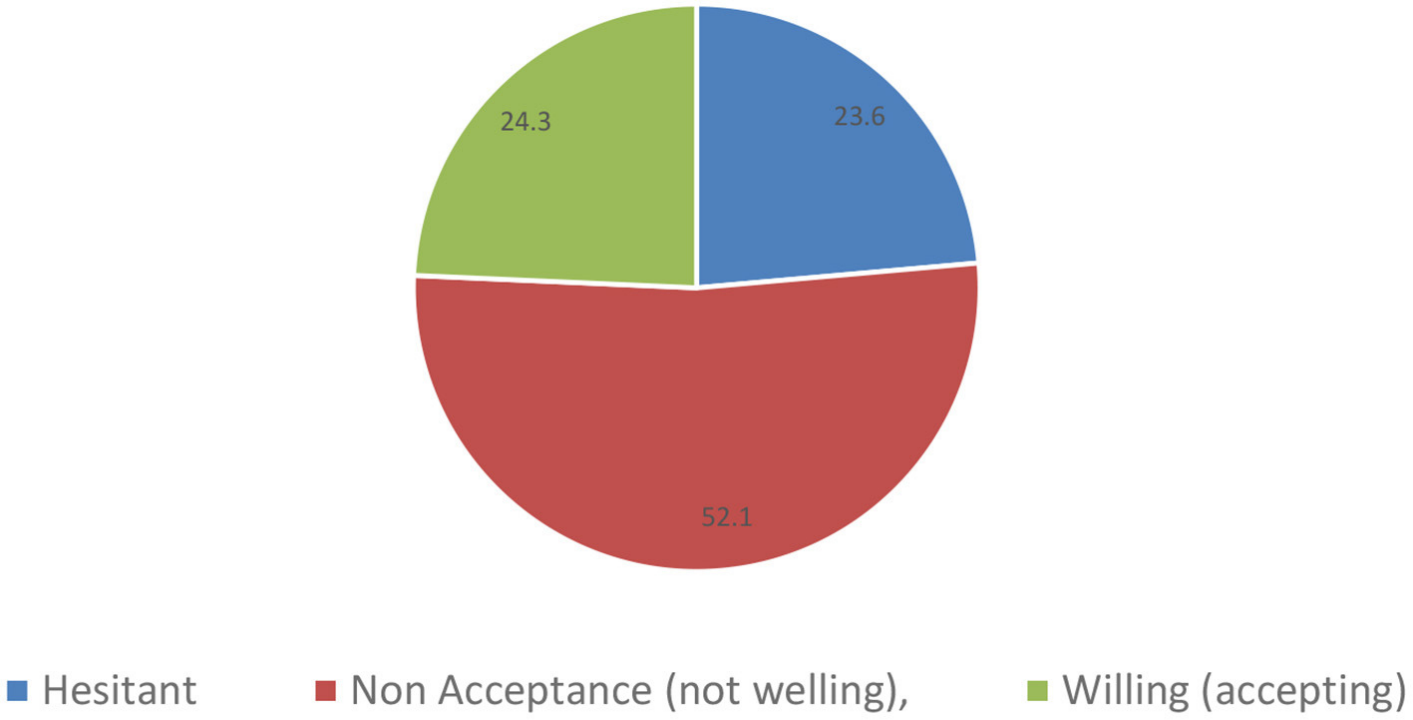
Intention of the physicians to get the COVID-19 vaccine. Shows the distribution of HCPs' status toward COVID-19 vaccine as “Hesitant,” “Non-Acceptance (not willing),” and “Willing (accepting)”.

The majority of physicians (227, 52.1%) were hesitant to take the vaccine, 236 (54.1%) had not yet decided on the preferred type of vaccine, 101 (23.2%) reported borderline (neutral) confidence in the Egyptian government to handle the pandemic, and 302 (96.3%) had no history of drug or food hypersensitivity (Figure 1; Table 3).

**Table 3:** The vaccine uptake; Intentions, Preference, and other related determinants among the studied physicians (*n* = 436).

**Variable**	**F (%)**
**Type of preferred vaccine** ^**#**^	
Chinese vaccine	26(6.0)
Pfizer vaccine	121(27.8)
Moderna vaccine	10(2.3)
AstraZeneca	31(7.1)
Russian vaccine	12(2.8)
Not decided yet	236(54.1)
**History of drug or food hypersensitivity**	
Yes	48 (11.0)
No	302(96.3)
Don't know	86(19.7)
**Confidence in the Egyptian government to handle the pandemic**	
Strongly disagree	93 (21.3)
Disagree	89(20.4)
Slightly disagree	46(10.6)
Borderline (neutral)	101(23.2)
Slightly agree (slight confidence)	44(10.1)
Agree (confident)	57(13.1)
Strongly agree	6(1.3)

# The known and approved vaccines during the time of data collection.

**Figure 2:**
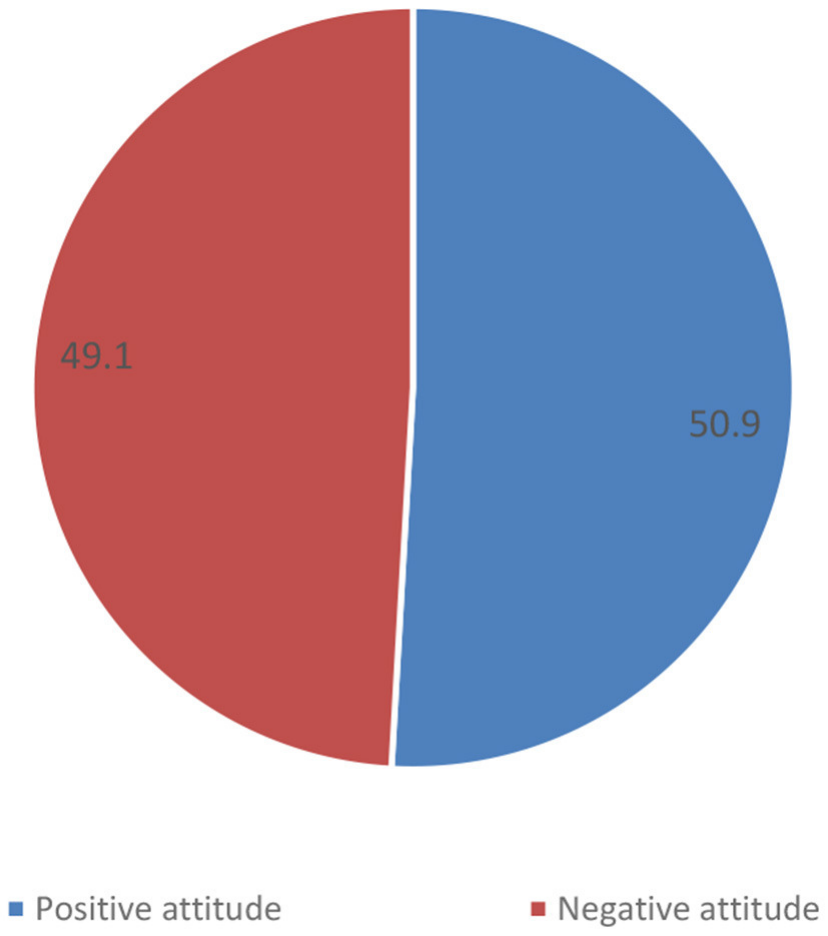
Attitude of the physicians regarding the COVID-19 vaccine. Shows the distribution of HCPs' status toward COVID-19 vaccine with regards to “Positive” and “Negative” attitudes.

In terms of the attitude of the physicians toward the COVID-19 vaccine, 222 (50.9%) showed positive attitudes, and 214 (49.1%) showed negative attitudes (Figure 2). There was no statistically significant (*p* > 0.05) relationship between physicians' attitudes toward COVID-19 vaccine uptake and any of the demographic variables studied (age, residence, sex, education, marital status, smoking, frontline work, and experience duration) (Table 1).

There was a statistically significant (*p* < 0.05) relationship between physicians' intentions to receive COVID-19 vaccinations and marital status, residence, whether the physicians were frontline, and perceived susceptibility. The highest willingness rates were 61 (57.5%) among rural physicians, 75 (70.7%) among infection-prone physicians, and 69 (65.1%) among non-frontline physicians (Table 4).

**Table 4:** Association between intentions toward COVID-19 vaccination uptake and some sociodemographic and health-related factors.

**Sociodemographic** **and health** **variables**	**Intentions toward COVID-19 vaccination uptake**			***X*^2^ test (*p*)**
	**Willing** **No=106 *F* (%)**	**Hesitated** **No=227** ***F* (%)**	**Not willing** **No=103 *F* (%)**	
**AGE group**				
20– <30	56(52.8%)	116(51.1%)	57(55.3%)	3.69
30 <40	32(30.2%)	81(35.7%)	37(35.9%)	(0.45)
40+	18(16.9%)	30(13.2%)	9(8.7%)	
**Gender**				
Male	46(43.3%)	76(33.4%)	30(29.3%)	5.1
Female	60(56.6%)	151(66.5%)	73(72.7%)	(0.07)
**Residence**				
urban	45(42.5%)	129(56.8%)	65(64.4%)	9.7
Rural	61(57.5%)	98(43.2%)	38(36.6%)	(0.01) ^*^
**Marital status**				
single	50(47.2%)	104(45.8%)	51(50.7%)	
married	55(51.9%)	119(52.4%)	45(44.6%)	9.54(0.046) ^*^
Divorced –widowed	1(0.9%)	4(1.8%)	7(6.7%)	
**Frontline worker**				
Yes	37(34.9%)	103(45.4%)	33(32.9%)	6.6
No	69(65.1%)	124(54.6%)	70(69.1%)	(0.037) ^*^
**Infected with COVID-19**				
yes	21(19.8%)	51(22.5%)	23(22.3%)	7.3
No	64(60.4%)	120(52.9 %)	45(43.6%)	(0.12)
Don't know	21(19.8%)	56(24.7%)	35(34.1%)	
**Susceptible to infection**				
Yes	75(70.7%)	187(82.4%)	74(73.0%)	9.94
No	9(8.5%)	6(2.6%)	8(7.7%)	(0.04) ^*^
Don't know	22(20.7%)	34(14.9%)	21(20.3%)	

* Significant at the 0.05 level (2-tailed). X^2^ test (chi-square test).

There was no statistically significant difference between the age groups and the Intentions toward COVID-19 vaccination uptake.

The main reasons for negative attitudes toward COVID-19 vaccination uptake were, in descending order, 328 (75.2%) preference for natural immunity, 312 (71.6%) mistrust of vaccine benefits, 305 (70%) concerns about commercial profiteering, and 255 (58.5%) concerns about unforeseen effects. There was a statistically significant relationship between all the physicians' intentions toward COVID-19 vaccination uptake and the entire four-attitude domains studied (Table 5).

**Table 5:** Frequency distribution of attitudes toward the vaccine association between intentions toward COVID 19 vaccination and attitudes toward vaccines.

	**Total** ***n* = 436** ***F* (%)**	**Intentions toward COVID 19 vaccination uptake**			**X^2^** **test (*p*)**
		**Willing** **No=106** ***F* (%)**	**Hesitated** **No=227 *F* (%)**	**Not willing** **No=103** ***F* (%)**	
**Mistrust of vaccine benefit mean±SD (2.92** **±1.03)**					67.2 (0.00) ^**^
Attitude					
Positive	124(28.4)	12(11.3%)	61(26.8%)	51(59.2%)	
Negative	312(71.6)	94(88.7%)	176(73.1%)	42(40.7%)	
**Worries about unforeseen effects mean±SD (2.92** **±1.03)**					4.32 (0.00) ^**^
Attitude					
Positive	181(41.5)	51(48.1%)	95(41.8%)	35(33.9%)	
Negative	255(58.5)	55(51.9%)	132(58.1%)	68(66.1%)	
**Concerns about commercial profiteering mean±SD (2.92** **±1.03)**					15.44 (0.00) ^**^
Attitude					
Positive	131(30.0)	47(44.4%)	63(27.8%)	21(20.3%)	
Negative	305(70.0)	59(55.6%)	164(72.2%)	82(79.6%)	
**Preference of natural immunity mean±SD (3.17** **±1.07)**					11.004 (0.004) ^*^
Attitude					
Positive	108(24.8)	38(35.8%)	53(23.3%)	17(16.5%)	
Negative	328(75.2)	68(64.2%)	174(76.7%)	86(83.5%)	

** Significant at the 0.01 level (2-tailed)/

* Significant at the 0.05 level (2-tailed).

X^2^ test (chi-square test).

There was a significant (*p* = 0.01) positive correlation between age and mistrust of the vaccine benefits, and an insignificant negative correlation between concerns about commercial profiteering and preference for natural immunity (Table 6).

**Table 6:** Correlation between age and attitude toward COVID-19 vaccine.

**Variables**	**Age(y)**	
	**R**	**P**
Mistrust of vaccine benefit	0.12	0.01^*^
Worries about unforeseen effects	0.078	0.104
Concerns about commercial profiteering	−0.089	0.063
Preference of natural immunity	−0.015	0.757

* Correlation is significant at the 0.05 level (2–tailed)./r correlation coefficient.

Multinomial logistic regression analysis was used to identify predictors of COVID-19 vaccine hesitancy and non-acceptance. The reasons for vaccination hesitancy and non-acceptance of vaccine uptake were revealed to be urban residence, concerns about future side effects, and vaccine mistrust, benefit, and preference for natural immunity were significant independent predictors of vaccine hesitancy (*p* = 0.004, 0.01, 0.00, and 0.03, respectively (Table 7).

**Table 7:** Multinomial regression analysis for the predictors of the COVID-19 vaccine uptake, hesitancy and non-acceptance.

**Intentions**		**B**	**Sig**.	**Exp(B)**	**95% Confidence interval for exp(B)**	
					**Lower bound**	**Upper bound**
**Vaccine hesitancy** ^a^						
	Mistrust of vaccine benefit	1.064	0.00^*^	0.345	0.242	492
	Worries about future side effects	0.351	0.01^*^	1.421	1.0931	0.846
	Commercial profiteering	0.119	0.39	1.127	0.856	1.483
	preference of natural immunity	0.333	0.03^*^	1.395	1.035	1.881
	Rural	0^b^	.	.	.	.
	Urban	0.761	0.004^**^	2.140	1.272	3.602
	Frontline (no)	0^b^	.	.	.	.
	Frontline (yes)	0.384	0.152	1.468	0.868	2.484
Non-acceptance of the vaccine uptake^a^						
	Mistrust of vaccine benefit	2.038	0.000^**^	0.130	0.083	204
	Worries about future side effects	0.501	0.002^*^	1.650	1.202	2.265
	Commercial profiteering	0.129	0.464	1.138	0.805	1.609
	preference of natural immunity	0.562	0.003^**^	1.754	1.209	2.544
	Rural	0^b^	.	.	.	.
	Urban	0.978	0.004^**^	2.660	1.363	5.192
	Frontline (no)	0^b^	.	.	.	.
	Frontline (yes)	−0.421–	0.236	0.656	0.327	1.316

a The reference category is: willing to the vaccine uptake.

b This parameter is set to zero because it is redundant.

** Significant at the 0.01 level (2-tailed)/

* Significant at the 0.05 level (2-tailed).

## Discussion

The COVID-19 vaccine was deemed the ideal solution for combating the existing pandemic, yet HCPs' vaccine hesitancy has been a challenge for healthcare leaders. Egypt has launched several vaccination programs, but the newness of the COVID-19 vaccination rollout has raised concerns about physicians' attitudes and acceptance of the vaccination. As a result, this novel study was carried out in Egypt to investigate this issue. During the second wave of the pandemic, this study was conducted right before the CDC and WHO approved all available vaccinations in Egypt and right before the vaccines were administered.

The majority of the studied physicians (227, 52.1%) were hesitant to take the vaccine, which was clearly higher than what was reported in other studies in different countries. In KSA 28.1% were unsure, while in America 31.6%, and in United Kingdome, and Portugal were hesitant (17, 21–24).

Less than 25% of the studied physicians were willing to accept the vaccine. Different rates were reported in different countries, all of which were higher than those in Egypt. Over time, with experience with actual vaccine administration and the current pandemic's time-varying death rates, COVID-19 vaccine willingness can change dramatically (21). For example, 88.6% was the median global acceptance rate from a survey of 19 countries, ranging from 59 to 75% in most Western countries (25). Specifically, the rates were 60% to 90% among physicians in Greece (February 2020), (26) 77.6% in France (March to July 2020), (27) 69% in KSA (November 2020), (28) 64.7% in America, (29) the figure was found at 36 and 57.6% in Singapore, and US (8, 23), 8% in Congo (March to April 2020), (9) 59% in South Africa (March to May 2021), (30) and nearly similar to other studies in Egypt (21, 27%) among Egyptian HCPs (11, 21).

This high vaccination hesitancy (VH) and low vaccination acceptance rate among HCPs in Egypt could be explained by the reported low and borderline or neutral levels of confidence in the Egyptian health care system, as well as the high prevalence of negative attitudes reported by more than 70% of physicians toward the uptake of COVID-19 vaccination, which was, in descending order, preference for natural immunity, mistrust of vaccine benefits, and concerns about commercial profiteering. VH is linked to negative attitudes about the SARS-CoV-2 vaccine, such as fears about safety and effectiveness, doubts about the need for vaccination, and preference for natural immunity (23, 25, 31).

Among the studied HCPs in Egypt, 103 (23.6%) were not willing to take the vaccine, which was higher than what was reported in other studies and lower than 41.0% in South Africa (March to May 2021) (30). For example, 10.8 to 25% of Americans, 20% of Canadians, 9% of Portuguese, and 7% of Saudis would not receive the vaccine (21–23, 32). Because of the extent of non-compliance, achieving herd immunity would be extremely difficult.

Although only 3.7% of HCPs had a history of drug or food hypersensitivity, approximately 24% of physicians were unwilling to uptake the vaccine. This figure is lower than that reported in a previous study of 40% of Egyptians (9), which could be explained by the fact that physicians had a higher level of medical education about the importance and effectiveness of the vaccine than the rest of the Egyptian community.

Vaccine willingness can change dramatically with time, experience with actual vaccine administration, and the current pandemic's time-varying morbidity and death rates (33). Physicians' acceptance of using the COVID-19 vaccine depends on the availability of the vaccine, the type of the vaccine, the degree of confidence in the healthcare system, and the vaccination policy. However all these determinants are changeable from time to time (34).

There was a statistically significant (*p* < 0.05) relationship between physicians' intentions to receive COVID-19 vaccinations and the physician's sex. However, most respondents (284, 65.1%) were women, in contrast with other studies which reported significantly lower acceptance among women (11, 17, 35).

A study in Bangladesh reported that participants living in urban areas were more than twice as likely to be aware of COVID-19 vaccination and willing to receive it (36). In contrast to our study, physicians who live in rural areas were significantly more likely to accept the vaccination. This can be explained by the fact that the population in rural areas has a poor practice for preventive measures, making physicians feel at increased risk for infection (37).

This study showed that age was found to be insignificantly associated with vaccination decisions. This was consistent with Fares et al., who found that the youngest age group had the highest uptake of the COVID-19 vaccine (21) This contradicted Grech et al., who found that the oldest age group had the highest uptake of the COVID-19 vaccine because they are the most vulnerable, and thus, more likely to accept the vaccine (38).

There was an insignificant relationship between physicians' attitudes toward COVID-19 vaccine uptake and all the studied demographic variables. In contrast to a study conducted in Bangladesh, participants' attitudes toward the COVID-19 vaccine were significant in terms of all demographic variables studied except perceived susceptibility and health status (36).

Our findings suggest that the most significant attitudinal barriers to receiving a COVID-19 vaccine are general distrust regarding vaccine benefits and safety and concerns about unforeseen side effects. This supports previous research that found low vaccine confidence and concerns about the novelty and safety of the COVID-19 vaccine to be significant attitudinal barriers to vaccine willingness (17).

The majority of respondents (71.6 %) did not believe in the benefits of the COVID-19 vaccine. This is consistent with another Egyptian study, which found that 79% of respondents did not trust received vaccine information (21). This was also similar to the findings of a study conducted in the United States, which found that a high percentage of HCPs did not trust information about COVID-19 and its severity provided by regulatory authorities and pharmaceutical companies for vaccine development and safety (17).

In the existing study, participants expressed a high level of concern about the COVID-19 vaccine's unforeseen effects, the percentage of which differed significantly between groups. This was supported by an Australian study by Dodd et al., which found that 36% of those who were hesitant to get the vaccine were concerned about its safety, compared to 11% of those who were willing to get the vaccination (39). Concerns about vaccination safety and effectiveness, as well as trial and testing duration, were common findings in many studies (40).

Assessing vaccine uptake predictors among HCPs is critical because it will enable health authorities and policymakers to target resources to maximize uptake. In this study, participants' willingness to administer COVID-19 vaccines was found to be significantly influenced by their income and years of experience. For diverse groups of HCPs who answered identical surveys in different regions of the world, the predictors were willingness to obtain influenza vaccinations years and people who classified themselves as having a high risk of severe COVID-19 infection (11).

Based on the reported maximum vaccine uptake, health officials must reassure the public that vaccine development adhered to all predetermined guidelines and that the process of developing the vaccine was not rushed. If the public believes that health officials are rushing a vaccine into production, this will erode public trust and exacerbate vaccine acceptance.

The most important way to ensure vaccine uptake is to provide convincing evidence that a SARS-CoV-2 vaccine has been rigorously tested, proven to be effective, and has not been rushed into production. Concerns about commercial profiteering are a significant barrier to vaccination uptake. Vaccine development and dissemination programs with more reassuring titles are more likely to gain public trust (41, 44).

By 28 April 2021, the COVID-19 mortality in Egypt reached 13,219, according to the Egyptian Ministry of Health and Population and WHO.^36.^ On the same day, the Egyptian Medical Syndicate reported that 492 Egyptian physicians had died of COVID-19 since the start of the pandemic (42), accounting for 3.7% (492/13,219) of COVID-19 mortalities in the country. The reported high negative attitudes and lack of willingness to vaccinate may lead to an exacerbation of the situation (45–47).

## Strength

The relatively large sample of physicians working in governmental healthcare settings in urban and rural areas represents physicians from Egypt's seven regions. The representation of both sexes, age groups, specialties, and proximity in dealing with COVID-19 patients.

## Limitation

The fact that this study was conducted exclusively online restricts the generalizability of the findings and may lead to selection bias. The study was conducted before COVID-19 vaccines were offered to HCPs in Egypt, so the acceptance rate may have altered once the vaccines were available.

## Conclusion

According to this study, Egyptian physicians were commonly hesitant to take the COVID-19 vaccine despite their susceptibility to the virus itself. There were statistically significant differences in the COVID-19 vaccination attitude and health status and perceived susceptibility. The high negative attitudes related to preference for natural immunity, mistrust of vaccine benefits, and concerns about commercial profiteering were significantly related to the widespread COVID-19 vaccination hesitancy and non-acceptance. Urban residence, concerns about future side effects, and vaccine mistrust, benefit, and preference for natural immunity were significant independent predictors of vaccine hesitancy and non-acceptance.

## Recommendations

As long as physicians' attitudes and perceptions of COVID-19 vaccines play an important role in the general population's vaccination behavior through consultation, we recommend that (1) This study's findings be shared with policymakers. (2) Policymakers should take these findings into account when planning and implementing public health intervention campaigns in Egypt to change negative vaccine attitudes and increase acceptance and uptake of COVID-19 vaccines to achieve herd immunity and control the pandemic. (3) Well-structured mass health education campaigns, advising on the significant implications for vaccine safety be implemented to reassure physicians and the public to maximize public uptake of the SARS- CoV-2 vaccine. (4) More research and interventions be conducted to address the various anti-vaccination beliefs that have been identified, as well as the best practices for reducing these negative beliefs.

## Data availability statement

The raw data supporting the conclusions of this article will be made available by the authors, without undue reservation.

## Ethics statement

The study methodology was approved by the Ethical Committee of Scientific Research, Faculty of Medicine, Benha University, No (RC.3.1.2021). All participants provided electronic informed written consent after clarification of the goals, data confidentiality, voluntary participation, and withdrawal.

## Author contributions

Study conception and design: SA and EA-E. Data collection: SA and HE. Analysis and interpretation of results: SA and JS. Draft manuscript preparation: SA, JS, EA-E, and HE. All authors contributed to the article and approved the submitted version.

## Conflict of interest

The authors declare that the research was conducted in the absence of any commercial or financial relationships that could be construed as a potential conflict of interest.

## Publisher's note

All claims expressed in this article are solely those of the authors and do not necessarily represent those of their affiliated organizations, or those of the publisher, the editors and the reviewers. Any product that may be evaluated in this article, or claim that may be made by its manufacturer, is not guaranteed or endorsed by the publisher.
